# Sow-Offspring Diets Supplemented with Probiotics and Synbiotics Are Associated with Offspring’s Growth Performance and Meat Quality

**DOI:** 10.3390/ijms24087668

**Published:** 2023-04-21

**Authors:** Qian Zhu, Md. Abul Kalam Azad, Haibo Dong, Chenjian Li, Ruixuan Li, Yating Cheng, Yang Liu, Yulong Yin, Xiangfeng Kong

**Affiliations:** 1 Key Laboratory of Agro-ecological Processes in Subtropical Region, Hunan Provincial Key Laboratory of Animal Nutritional Physiology and Metabolic Process, Institute of Subtropical Agriculture, Chinese Academy of Sciences, Changsha 410125, China; zhuqian@isa.ac.cn (Q.Z.); azad.fpe@gmail.com (M.A.K.A.); dhaibo89@gmail.com (H.D.); 2021807204@stu.njau.edu.cn (C.L.); raphaell2131@outlook.com (R.L.); chengyating20@mails.ucas.ac.cn (Y.C.); liuyang@stu.njau.edu.cn (Y.L.); yinyulong@isa.ac.cn (Y.Y.); 2 National Engineering Laboratory for Pollution Control and Waste Utilization in Livestock and Poultry Production, Institute of Subtropical Agriculture, Chinese Academy of Sciences, Changsha 410125, China; 3 College of Advanced Agricultural Sciences, University of Chinese Academy of Sciences, Beijing 100049, China; 4 Research Center of Mini-Pig, Huanjiang Observation and Research Station for Karst Ecosystems, Chinese Academy of Sciences, Huanjiang 547100, China

**Keywords:** growth performance, carcass traits, meat quality, probiotics, synbiotics, Bama mini-pigs

## Abstract

Probiotics and synbiotics supplementation have been shown to play potential roles in animal production. The present study aimed to evaluate the effects of dietary probiotics and synbiotics supplementation to sows during gestation and lactation and to offspring pigs (sow-offspring) on offspring pigs’ growth performance and meat quality. Sixty-four healthy Bama mini-pigs were selected and randomly allocated into four groups after mating: the control, antibiotics, probiotics, and synbiotics groups. After weaning, two offspring pigs per litter were selected, and four offspring pigs from two litters were merged into one pen. The offspring pigs were fed a basal diet and the same feed additive according to their corresponding sows, representing the control group (Con group), sow-offspring antibiotics group (S-OA group), sow-offspring probiotics group (S-OP group), and sow-offspring synbiotics group (S-OS group). Eight pigs per group were euthanized and sampled at 65, 95, and 125 d old for further analyses. Our findings showed that probiotics supplementation in sow-offspring diets promoted growth and feed intake of offspring pigs during 95–125 d old. Moreover, sow-offspring diets supplemented with probiotics and synbiotics altered meat quality (meat color, pH_45min_, pH_24h_, drip loss, cooking yield, and shear force), plasma UN and AMM levels, and gene expressions associated with muscle-fiber types (*MyHCI*, *MyHCIIa*, *MyHCIIx*, and *MyHCIIb*) and muscle growth and development (*Myf5*, *Myf6*, *MyoD*, and *MyoG*). This study provides a theoretical basis for the maternal-offspring integration regulation of meat quality by dietary probiotics and synbiotics supplementation.

## 1. Introduction

Pork is the most consumed meat worldwide and accounts for 37% of all meat, more than beef or chicken [1]. In recent years, the demand for high-quality meat has been increasing dramatically. As a result, growing studies are dedicated to producing delicious meat with higher nutritional values in order to meet consumer demand. Skeletal muscle accounts for about 40−60% of the mammalian body weight (BW) [2]. The skeletal muscle is a highly heterogeneous tissue type, which is closely related to economic traits, such as muscle growth and development and meat quality in livestock and poultry production [2]. Pork quality can be affected by numerous factors, and the type and composition of muscle fiber is one of the crucial factors. Muscle-fiber characteristics are associated with numerous meat quality traits, including pH, drip loss, meat color, tenderness, and intramuscular fat content [3]. In addition, the size, number, and type of muscle fibers are closely interrelated and implicated in muscle-fiber characteristics [4]. The primary myofibers develop between 25 and 50 days of gestation and act as a template for forming secondary fibers that are completed between 80 to 90 days of gestation [5]. Furthermore, muscle development undergoes significant changes during ontogenesis [6]. Hence, improving the growth and development of skeletal muscle is a vital strategy to improve the productivity and quality of pork.

During gestation and lactation, the nutritional and health status of the sow has pivotal effects on their offspring’s pre- and post-natal growth and development. The placenta and milk are the determining factors that could affect intrauterine fetal development and postpartum neonatal growth and development. Moreover, the fetal and neonatal are the most vital periods for skeletal muscle development. Furthermore, the development of post-natal muscle fibers is determined by the composition and types of fetal and neonatal muscle fibers [7]. Therefore, regulation of maternal nutrition and health plays a pivotal role in the skeletal muscle performance of offspring pigs.

Feed additives are always used to improve productivity and meat quality in swine production. For example, prebiotics, probiotics, and synbiotics have been studied extensively as feed additives in swine production, which have shown significant impacts on microbial community composition in the short and long term [8]. In the past few years, numerous studies have been conducted on the beneficial effects of probiotics and prebiotics in animals. Shin et al. [9] revealed that probiotics supplementation has beneficial effects on pigs, including improving growth performance, promoting nutrient digestion, absorption, and utilization, modulating intestinal microbiota, as well as ameliorating gut health complications. Additionally, a previous study illustrated that oral administration of β-glucan could improve the duodenal villi dimensions, splenic lymphoid diameter, muscular fiber diameter, and muscular glycogen areas in New Zealand white and APRI rabbits [10]. Dietary supplementation of *Saccharomyces cerevisiae* provided beneficial effects on growth performance and profitability in rabbits [11]. Abd EI-Aziz et al. [12] highlighted that fructo-oligosaccharide supplementation in drinking water enhances growth and carcass traits by improving the hematobiochemical parameters and antioxidant status and reducing cecal pathogenic bacteria in two different rabbit breeds. Moreover, our previous study indicated that sows and their offspring’s (sow-offspring) diets supplemented with probiotics or synbiotics could alter microbiota composition by increasing the abundance of beneficial bacteria (i.e., *Bifidobacterium* and *Lactobacillus*), decreasing potentially harmful bacteria (i.e., *E.coli*), and enhancing the immune and antioxidant capacity in offspring pigs [13]. In addition, these feed additives have also been found to be an effective strategy for improving pork quality by improving meat redness and tenderness, increasing meat protein, and decreasing meat drip loss [14]. However, a few studies focused on whether sow-offspring diets supplemented with probiotics or synbiotics could improve the growth performance and meat quality in offspring pigs.

Thus, we hypothesized that sow-offspring probiotics and synbiotics supplementation could improve offspring pigs’ growth performance and meat quality. Therefore, the present study was conducted to explore the effects of dietary probiotics and synbiotics supplementation in sow-offspring diets on growth performance and meat quality in offspring pigs at different time points (65, 95, and 125 d old) after weaning. Thus, we examined related indicators and revealed the possible mechanism from the aspects of the metabolism of the body and gene expression related to the muscle-fiber type and muscle growth. Moreover, this study could also provide the theoretical basis for improving pork quality.

## 2. Results

### 2.1. Growth Performance of Offspring Pigs

At 125 d old, probiotics supplementation increased the BW, average daily gain (ADG), and average daily feed intake (ADFI) of offspring pigs, whereas synbiotics supplementation decreased these three indexes in comparison with the control group (Con group, *p* < 0.05). Furthermore, the ADG of offspring pigs was higher, though the feed-gain ratio (F/G) was lower in the sow-offspring probiotics group (S-OP group) than in the sow-offspring antibiotics group (S-OA group) at 125 d old (*p* < 0.05). However, the BW at 125 d old and ADG, F/G, and ADFI during 96–125 d old were lower (*p* < 0.05) in the sow-offspring synbiotics group (S-OS group) than in the S-OA group (Table 1).

### 2.2. Carcass Traits of Offspring Pigs

Compared with the Con group, all indicators, including the backfat thickness, carcass weight, fat percentage, bone percentage, muscle percentage, leaf-fat ratio, and the loin-eye muscle area, were not affected by probiotics supplementation at 65 d old, as well as the carcass weight, fat percentage, bone percentage, and leaf-fat percentage at 95 d old (*p* > 0.05). However, at 125 d old, the backfat thickness, carcass weight, fat percentage, and leaf-fat ratio in the S-OP group and bone percentage in the S-OS group were higher, whereas muscle percentage and loin-eye muscle area in the S-OS group were lower than in the Con group (*p* < 0.05). Moreover, the fat percentage during 65–125 d old, leaf-fat ratio at 95 and 125 d old, and muscle percentage at 65 and 95 d old were higher in the S-OP group than in the S-OA group (*p* < 0.05). The backfat thickness was lower, though the muscle percentage and loin-eye muscle area were higher in the S-OS group than in the S-OA group at 65 d old (*p* < 0.05). Moreover, the backfat thickness and muscle percentage were not changed (*p* > 0.05), though the carcass weight and loin-eye muscle area were decreased (*p* < 0.05) in the S-OS group in comparison with the S-OA group at 125 d old (Table 2).

### 2.3. Meat Quality of Offspring Pigs

Meat quality parameters are presented in Table 3. Compared with the Con group, at 65 d old, lightness (L*) value and drip loss of the *longissimus dorsi* muscle (LDM) were reduced in the S-OA, S-OP, and S-OS groups, whereas the pH value of the LDM at 24 h (pH_24h_) was elevated in the S-OP group, as well as cooking yield and shear force of the LDM in the S-OS group (*p* < 0.05). The L* value of the LDM was reduced in the S-OA and S-OP groups, as well as drip loss in the S-OP and S-OS groups at 95 d old in comparison with the Con group (*p* < 0.05). The redness (a*) value and cooking yield of the LDM were increased, whereas the pH_24h_ was decreased in the S-OA group in comparison with the Con group (*p* < 0.05). At 125 d old, shear force and drip loss of the LDM were reduced in the S-OA, S-OP, and S-OS groups compared with the Con group (*p* < 0.05). The yellowness (b*) value of the LDM was reduced in the S-OA group, though elevated in the S-OS group compared with the Con group (*p* < 0.05). Compared with the Con group, the pH value at 45 min (pH_45min_) of the LDM in the S-OS group was elevated (*p* < 0.05), as well as pH_24h_ in the S-OA group. Moreover, compared with the S-OA group, the pH_24h_ was elevated but the cooking yield was reduced at 95 d old, whereas the b* value was elevated but pH_24h_ was reduced at 125 d old in the S-OP and S-OS groups (*p* < 0.05). Moreover, drip loss, cooking yield, and shear force of the LDM at 65 d old were increased, while the a* value of the LDM at 95 d old was reduced in the S-OS group compared with the S-OA group (*p* < 0.05).

### 2.4. Amino Acids Profile in the Skeletal Muscle of Offspring Pigs

Probiotics supplementation in sow-offspring diets did not affect the amino acids profile in the LDM of offspring pigs at 65 d old in comparison with the Con group (*p* > 0.05). Synbiotics supplementation increased proline (Pro) content, whereas reduced the content of aspartic acid (Asp), glycine (Gly), and leucine (Leu) in the LDM of offspring pigs at 65 d old in comparison with the Con group (*p* < 0.05). The content of alanine (Ala), Asp, and Leu were elevated, whereas Pro and histidine (His) were reduced in the LDM of offspring pigs in all three treated groups in comparison with the Con group at 95 d old (*p* < 0.05). The content of glutamic acid (Glu), threonine (Thr), His, serine (Ser), and tyrosine (Tyr) in the S-OA group, Glu, Thr, Ala, Asp, arginine (Arg), and the total amino acids (TAA) in the S-OP group, and His, Ala, and Pro in the S-OS group were elevated in comparison with the Con group at 125 d old (*p* < 0.05). In addition, compared with the S-OA group, the content of Asp, Gly, Leu, lysine (Lys), valine (Val), essential amino acids (EAA), flavor amino acids (FAA), and TAA at 65 d old, and Ser at 125 d old were decreased (*p* < 0.05) in the S-OS group, as well as Ala, isoleucine (Ile), and Thr at 65 d old in the S-OP and S-OS groups (Table 4).

In Table 5, in comparison with the Con group, the content of Pro and phenylalanine (Phe) in the *psoas major* muscle (PMM) were increased in the S-OA, S-OP, and S-OS groups, as well as Glu, Gly, Ser, Arg, Val, nonessential amino acids (NEAA), FAA, and TAA in the S-OA group at 65 d old (*p* < 0.05). Moreover, the content of Tyr was elevated, though crude protein (CP), Asp, and Lys were reduced in the S-OS group (*p* < 0.05). In comparison with the Con group, the content of Ala was reduced in the S-OP and S-OS groups, as well as His and Thr in the S-OS group at 95 d old (*p* < 0.05). At 125 d old, the content of His in all three treated groups, Pro and Tyr in the S-OP and S-OS groups, and Thr and NEAA in the S-OS group were increased, whereas Tyr was decreased in the S-OA group in comparison with the Con group (*p* < 0.05). In addition, the content of Pro, Tyr, and NEAA at 125 d old was increased, while Ala, Glu, Gly, Ser, Val, NEAA, FAA, and TAA at 65 d old were reduced in the S-OP and S-OS groups in comparison with the S-OA group (*p* < 0.05).

### 2.5. Plasma-Free Amino Acids Concentrations of Offspring Pigs

Compared with the Con group, the concentrations of citrulline (Cit) and β-aminoisobutyric acid (β-AiBA) in the S-OA and S-OS groups, Thr, Pro, and α-amino-n-butyric acid (α-ABA) in the S-OA group, and His and hydroxy-lysine (Hylys) in the S-OP group were increased, whereas the hydroxy-proline (Hypro) in all three treated groups, cystathionine (Cysthi) in the S-OA group, Leu, Ala, and Tyr in the S-OP group, and Ile, Gly, sarcosine (Sar), and taurine (Tau) in the S-OA and S-OP groups were decreased (*p* < 0.05) at 65 d old. Moreover, the concentrations of Cysthi and γ-amino-n-butyric acid (γ-ABA) in the S-OS group were decreased (*p* < 0.05) compared with the Con, S-OA, and S-OP groups (Table 6).

At 95 d old, compared with the Con group, the concentration of β-AiBA was elevated, whereas the α-aminoadipic acid (α-AAA), α-ABA, β-alanine (β-Ala), γ-ABA, Cit, Ile, Leu, and Tau were reduced in the S-OA, S-OP, and S-OS groups (*p* < 0.05). The concentrations of His in the S-OA and S-OP groups, ethanolamine (EOHNH_2_) in the S-OP and S-OS groups, Ala in the S-OA group, carnosine (Car) in the S-OP group, and anserine (Ans) in the S-OS group were reduced, whereas the Sar in the S-OA group, EOHNH_2_, and 1-methyl-histidine (1Mehis) in the S-OP and S-OS groups, and Cysthi and Hylys in the S-OP group were elevated (*p* < 0.05). Furthermore, the concentration of ornithine (Orn) was elevated (*p* < 0.05), whereas 1Mehis and EOHNH_2_ were reduced (*p* < 0.05) in the S-OP and S-OS groups than in the S-OA group (Table 6).

At 125 d old, the concentrations of Orn, Ans, and methionine (Met) in the S-OA and S-OP groups, Leu, Val, and α-ABA in the S-OA and S-OS groups, Ala, Arg, Lys, Gly, Pro, Ser, Tau, Thr, Tyr, β-Ala, and β-AiBA in the S-OA group, and Ile and Cysthi in the S-OS group were increased, whereas the Orn in all treated groups, Glu in the S-OA and S-OS groups, Ser in the S-OP and S-OS groups, His and Asp in the S-OP groups, and EOHNH_2_ and β-AiBA in the S-OS group were decreased in comparison with the Con group (*p* < 0.05). Moreover, the concentrations of Ala, Arg, Ans, Gly, Ser, Met, Tyr, Pro, Thr, Tau, and β-AiBA were reduced (*p* < 0.05) in the S-OP and S-OS groups in comparison with the S-OA group (Table 6).

### 2.6. Plasma Biochemical Parameters of Offspring Pigs

Plasma biochemical parameters of offspring pigs are shown in Table 7. At 65 d old, compared with the Con group, the concentrations of total protein (TP) and ammonia (AMM) were increased in the S-OA and S-OP groups (*p* < 0.05). The alkaline phosphatase (ALP) activity was increased in the S-OP and S-OS groups, whereas it was decreased in the S-OA group compared with the Con group (*p* < 0.05). In addition, the urea nitrogen (UN) concentration was increased in the S-OP group in comparison with the Con group (*p* < 0.05). Moreover, the ALP activity was increased in the S-OP and S-OS groups in comparison with the S-OA group at 65 d old (*p* < 0.05). The alanine aminotransferase (ALT) activity in the S-OS group was increased, whereas the UN concentration in all three treated groups, and the TP and AMM concentrations in the S-OP group were reduced in comparison with the Con group at 95 d old (*p* < 0.05). At 125 d old, the AMM concentration was reduced in all three treated groups in comparison with the Con group (*p* < 0.05). In addition, the ALP activity and UN concentration at 65 and 125 d old were elevated (*p* < 0.05) in the S-OP group in comparison with the S-OA group. However, at 95 d old, the TP and UN concentrations were reduced in the S-OP group when compared with the other three treatment groups (*p* < 0.05). In addition, synbiotics supplementation increased the ALT activity at 65 and 95 d old, as well as the ALP activity at 65 d old and the TP concentration at 125 d old, though it decreased the ALP and aspartate aminotransferase (AST) activities at 95 d old, as well as AMM concentration at 65 d old compared with the S-OA group (*p* < 0.05).

### 2.7. mRNA Expression of Genes Associated with Muscle-Fiber Type and Muscle Growth in Skeletal Muscles of Offspring Pigs

**Figure 1:**
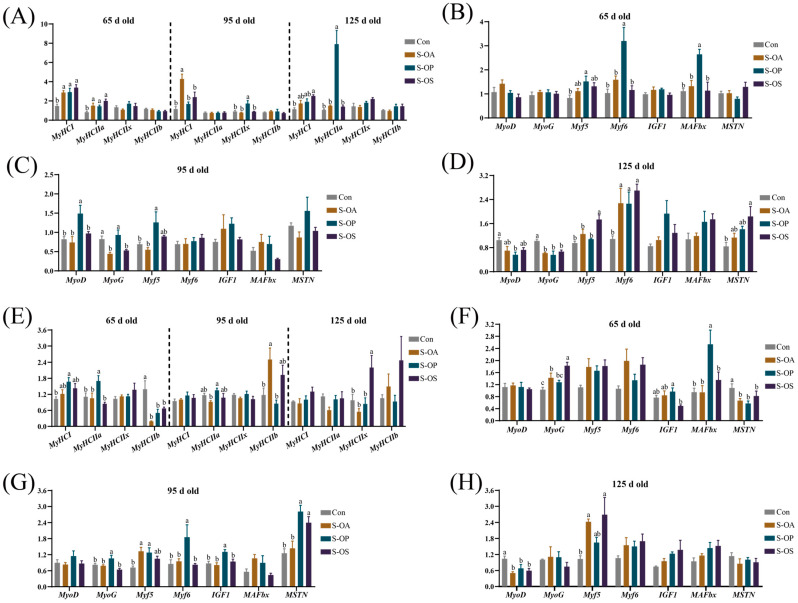
Effects of probiotics and synbiotics supplementation in sow-offspring diets on mRNA expressions of myosin heavy chain (MyHC) isoforms and myogenic regulatory factors (MRFs) in the skeletal muscle of offspring pigs at 65, 95, and 125 d old. (**A**) and (**E**) are mRNA expressions of MyHC isoforms in the *longissimus doris muscle* (LDM) and *psoas major* muscle (PMM), respectively. (**B**–**D**) and (**F**−**H**) are mRNA expressions of MRFs in the LDM and PMM, respectively. ^a–c^ Different letters mean significant differences (*p* < 0.05). *MyHCI*, myosin heavy chain I; *MyHCIIa*, myosin heavy chain IIa; *MyHCIIb*, myosin heavy chain IIb; *MyHCIIx*, myosin heavy chain IIx; *MyoD*, myogenic differentiation factor; *MyoG*, myogenin; *Myf5*, myogenic factor 5; *Myf6*, myogenic factor 6; *IGF1*, insulin-like growth factor 1; *MAFbx*, muscle atrophy Fbox-1 protein; *MSTN*, myostatin. Con group: sow and offspring pigs fed with a basal diet; S-OA group: sow and offspring pigs fed with antibiotics; S-OP group: sow and offspring pigs fed with probiotics; S-OS group: sow and offspring pigs fed with synbiotics. The replicates per group at 65 d old were 8. The replicates of the Con, S-OA, S-OP, and S-OS groups at 95 d old were 8, 8, 8, and 7, respectively. The replicates of the Con, S-OA, S-OP, and S-OS groups at 125 d old were 8, 5, 6, and 6, respectively.

Compared with the Con group, the expressions of myosin heavy chain I (*MyHCI*) and myosin heavy chain IIa (*MyHCIIa*) were upregulated in the LDM of offspring pigs in the S-OA, S-OP, and S-OS groups at 65 d old (*p* < 0.05). Antibiotics supplementation in sow-offspring diets upregulated the *MyHCI* expression, and probiotics supplementation upregulated myosin heavy chain IIx (*MyHCIIx*) expression in the LDM of offspring pigs at 95 d old (*p* < 0.05). The *MyHCIIa* expression in the LDM of offspring pigs was upregulated in the S-OP group in comparison with the Con, S-OA, and S-OS groups, and *MyHCI* expression in the LDM of offspring pigs was upregulated in the S-OS group in comparison with the Con group at 125 d old (*p* < 0.05) (Figure 1A). At 65 d old, the myogenic factor 5 (*Myf5*) expression was upregulated in the S-OP group compared with the Con group, while muscle atrophy Fbox-1 protein (*MAFbx*) and myogenic factor 6 (*Myf6*) expressions in the LDM of offspring pigs were upregulated in the S-OP group in comparison with the Con, S-OA, and S-OS groups (*p* < 0.05) (Figure 1B). At 95 d old, the expressions of myogenic differentiation factor (*MyoD*) and *Myf5* in the LDM of offspring pigs were upregulated in the S-OP group in comparison with the Con and S-OA groups, whereas myogenin (*MyoG*) was downregulated in the S-OA and S-OS groups in comparison with the Con and S-OP groups (*p* < 0.05) (Figure 1C). At 125 d old, the *Myf6* expression in the S-OA, S-OP, and S-OS groups and myostatin (*MSTN*) and *Myf5* expressions in the S-OS group were upregulated, whereas *MyoG* expression was downregulated in the S-OA, S-OP, and S-OS groups compared with the Con group (*p* < 0.05). Moreover, the *MyoD* expression in the LDM of offspring pigs was downregulated in the S-OP group compared with the Con group (*p* < 0.05) (Figure 1D).

In the PMM, the expression of *MyHCI* was upregulated in the S-OP group compared with the Con group, while *MyHCIIa* was upregulated in the S-OP group in comparison with the Con, S-OA, and S-OS groups; moreover, myosin heavy chain IIb (*MyHCIIb*) was downregulated in all three treated groups compared with the Con group at 65 d old (*p* < 0.05). At 95 d old, the *MyHCIIa* expression was upregulated in the S-OP group compared with the S-OA group, and the *MyHCIIb* expression was upregulated in the S-OA group in comparison with the Con and S-OP groups (*p* < 0.05). In addition, the *MyHCIIx* expression was upregulated in the S-OS group compared with the Con, S-OA, and S-OP groups at 125 d old (*p* < 0.05) (Figure 1E). At 65 d old, the *MyoG* expression was upregulated in the S-OA and S-OP groups, whereas *MSTN* was downregulated in the S-OA, S-OP, and S-OS groups compared with the Con group (*p* < 0.05). Moreover, the *MAFbx* expression was upregulated in the S-OP group compared with the Con, S-OA, and S-OS groups, while insulin-like growth factor 1 (*IGF1*) expression was upregulated in the S-OP group compared with the S-OS group (*p* < 0.05) (Figure 1F). As presented in Figure 1G, at 95 d old, the expressions of *MyoG*, *Myf6*, and *IGF1* in the S-OP group were upregulated compared with the Con, S-OA, and S-OS groups, while the *Myf5* in the S-OA and S-OP groups was upregulated compared with the Con group and *MSTN* in the S-OP and S-OS groups was upregulated compared with the Con and S-OA groups (*p* < 0.05). At 125 d old, the expression of *Myf5* was upregulated in the S-OA and S-OS groups, whereas *MyoD* was downregulated in the S-OA, S-OP, and S-OS groups compared with the Con group (*p* < 0.05) (Figure 1H).

## 3. Discussion

Growing interest has been given to the positive effects of probiotics and synbiotics, including promoting growth and development, improving gut health, boosting the immune system, and preventing diseases in humans and animals. Our previous study demonstrated that maternal diets supplemented with probiotics and synbiotics have beneficial roles in improving piglet performance [15]. However, it is poorly known whether sow-offspring diets supplemented with probiotics and synbiotics could have positive effects on the growth performance and meat quality of the offspring pigs. In the present study, our findings demonstrated that sow-offspring diets supplemented with probiotics and synbiotics have beneficial effects on meat quality, manifested in the improvement of sensory indexes (including tenderness, water-holding capacity, and meat flavor), alteration of plasma metabolites related to nitrogen metabolism, and gene expression associated with muscle-fiber types and muscle growth. In addition, probiotics supplementation in sow-offspring diets have better effects on improving growth performance and the meat quality of offspring pigs than antibiotics.

Generally, probiotics are live or dynamic microbes that are beneficial to the host’s health. Previous studies have demonstrated that probiotics and synbiotics play a vital role in improving the growth performance of weaned piglets [16] and growing-finishing pigs [17] and enhancing weaning BW gain [18]. In the present study, the BW, ADG, and ADFI of offspring pigs were significantly elevated in the S-OP group during 95–125 d old; however, these indicators had opposite changes in the S-OS group. These findings suggest that probiotics supplementation in sow-offspring diets could promote piglet feed intake and thus influence the growth of offspring pigs during 95–125 d old, which may be related to the improving effects of probiotics on the growth performance of offspring pigs. Several studies have shown that probiotics have positive effects on feed efficiency and BW gain [19,20,21]. However, synbiotics supplementation had an adverse effect on the growth performance of offspring pigs during 95–125 d old in this study. These differences may be related to the types of probiotics and prebiotics and their combination. A previous study also reported that there was no interaction between dietary supplementation of probiotics and xylo-oligosaccharides (XOS) concerning growth performance in weanling piglets [22]. However, fructo-oligosaccharide combined with *Lactobacillus plantarum* ZLP001 as a potential synbiotics showed synergistic effects [23].

Backfat thickness is positively correlated with the eating quality traits of meat, such as flavor, juiciness, and overall acceptability [24]. Our results showed that sow-offspring diets supplemented with three additives (antibiotics, probiotics, and synbiotics) significantly decreased the backfat thickness, and dietary antibiotics supplementation significantly decreased the fat percentage and leaf-fat ratio at 95 d old. However, dietary probiotics supplementation significantly increased the loin-eye muscle area and muscle percentage of offspring pigs at 95 d old. Moreover, our results showed that the loin-eye muscle area and muscle percentage of offspring pigs were significantly decreased in the S-OS group at 125 d old. These findings indicate that dietary antibiotics, probiotics, and synbiotics have a negative effect on fat deposition at 95 d old. Furthermore, probiotics and synbiotics supplementation significantly increase muscle mass at 125 d old. Liu et al. [25] also indicated that combining dietary multi-strain probiotics and *Castanea crenata* shell extract can increase the loin-eye muscle area of finishing pigs. In addition, sow-offspring diets supplemented with probiotics significantly increased the fat percentage, backfat thickness, and leaf-fat ratio of offspring pigs at 125 d old in the present study. These results suggest that dietary probiotics could enhance fat deposition, and then improve pork quality.

The physicochemical characteristics of meat affect its appearance and sensory quality [26]. Drip loss, color, pH, tenderness, and taste of pork are the most critical parameters for consumers [27]. Drip loss could change the chemical composition and affect the tenderness of meat [28]. Shear force is particularly important when assessing the value of meat intended for culinary purposes and is also an indicator of meat tenderness [29]. The pH value is a factor that has a direct influence on the technological properties of meat. A previous study indicated that the eating quality of meat is most desirable at intermediate pH (5.4–6.0) [30]. In the present study, the pH_24h_ value ranged from 5.46 to 5.62 in the LDM, suggesting normal glycolysis and glycogenolysis progress in the four treatment groups [31]. The declining rate of muscle pH is highly relevant to the drip loss and shear force of meat. In the present study, dietary probiotics supplementation significantly increased pH_24h_ at 65 d old, while synbiotics significantly increased pH_45min_ at 125 d old. However, probiotics and synbiotics supplementation significantly decreased the drip loss in the LDM at three different days of age in comparison with the Con group. These findings suggest that sow-offspring diets supplemented with probiotics and synbiotics could decrease the risk for PSE meat (pale, soft, and exudative). A recent study also showed that dietary multi-strain probiotics and *Castanea crenata* shell extract can reduce drip loss in finishing pigs [25]. In addition, our results also demonstrated a significant decrease in the shear force of LDM at 125 d old when supplemented with probiotics and synbiotics. These findings suggest that probiotics and synbiotics supplementation in sow-offspring diets may attenuate pH decline and positively affect the postmortem water-holding capacity and tenderness of the LDM.

Meat color is a critical indicator in evaluating muscular appearance, influencing consumers’ purchase decisions as it is deemed a visual measure of freshness and quality [32]. Cooking yield reflects the water loss during cooking. In the present study, the LDM lightness value of offspring pigs was significantly decreased at 65 d old when sow-offspring diets were supplemented with antibiotics, probiotics, and synbiotics, as well as at 95 d old when supplemented with antibiotics and probiotics; moreover, antibiotics supplementation significantly increased redness value and cooking yield at 95 d old, and synbiotics supplementation significantly increased cooking yield at 65 d old. These findings indicate that antibiotics, probiotics, and synbiotics could improve meat color to some extent when those were supplemented in sow-offspring diets. A previous study reported that *Bacillus subtilis* and *Clostridium butyricum* supplementation in sow diets increased meat color scores and redness [33]. Moreover, antibiotics and synbiotics supplementation could decrease the cooking loss of muscle.

Pork is the major animal protein source for humans, and amino acids composition determines muscle protein quality [34]. We found that sow-offspring diets supplemented with synbiotics significantly decreased the CP content of the PMM at 65 d old, suggesting that synbiotics have a negative effect on the protein accumulation of PMM. Meat with high-quality protein contains all EAA required by the mammalian body [35]. Several studies demonstrated that Lys, Arg, Asp, and Glu in meat are considered precursors for flavoring substances formation [36]. Our findings clarified that probiotics and synbiotics supplementation in sow-offspring diets significantly increased the Asp, Arg, and Glu content in the LDM at different stages of age. Histidine is a kind of EAA for infants and children [37]. In our study, at 125 d old, synbiotics supplementation significantly increased the His content in the LDM, as well as that in the PMM when probiotics and synbiotics were supplemented in sow-offspring diets. A previous study showed that Glu and Asp present a pleasant fresh taste, and Gly, Ala, and Ser present a sweet taste [38]. In the present study, dietary probiotics and synbiotics supplementation significantly enhanced the Ala content in the LDM at 95 and 125 d old, as well as Pro and Phe at 65 d old and Pro and Tyr at 125 d old in the PMM of offspring pigs. These findings suggest that sow-offspring diets supplemented with probiotics and synbiotics could alter the amino acid composition and then improve the meat flavor. A previous study demonstrated that diets supplemented with *Clostridium butyricum* could improve duck meat flavor by changing the FAA content [39]. Furthermore, significantly higher content of Glu, Gly, Ser, Arg, Val, NEAA, FAA, and TAA were noted in the PMM when sow-offspring diets were supplemented with antibiotics, indicating that antibiotics supplementation could improve meat flavor and nutritional value at 65 d old.

Free amino acids in plasma participate in muscle protein synthesis [40] and are essential in reflecting muscle nutrition metabolism. Branched-chain amino acids, including Leu, Ile, and Val, play important roles in energy homeostasis and nutritional metabolism [41]. Furthermore, Tau plays an important role in energy metabolism in the skeletal muscle, heart, liver, and adipose tissues [42]. The plasma concentrations of Leu, Ile, and Tau were significantly decreased at 65 d old when sow-offspring diets were supplemented with probiotics, as well as at 95 d old when sow-offspring diets were supplemented with probiotics and synbiotics in the present study. These findings suggest that dietary probiotics and synbiotics supplementation in sow-offspring diets are beneficial to muscle energy homeostasis and metabolism. A recent study demonstrated that Ser and Gly are essential for skeletal muscle regeneration [43]. Our findings showed that sow-offspring diets supplemented with probiotics and synbiotics significantly reduced the plasma Orn and Ser concentrations at 125 d old, indicating that these feed additives may be beneficial for skeletal muscle regeneration. In addition, sow-offspring diets supplemented with probiotics and synbiotics significantly reduced the plasma concentrations of γ-ABA, α-AAA, α-ABA, and β-Ala at 95 d old. Overall, the findings suggest that dietary probiotics and synbiotics supplementation in sow-offspring diets could promote AA deposition in the muscle of offspring pigs. However, further investigations are needed to reveal the underlying mechanism.

The nutritional status of animals could be reflected by the plasma biochemical parameters [44]. The ALP activity reflects the growth performance and is positively correlated with ADG and calcium and phosphorus utilization rate [45]. Our study demonstrated that sow-offspring diets supplemented with probiotics and synbiotics significantly increased the plasma ALP activity at 65 d old. However, the ADG of offspring pigs did not significantly change, which may be due to the supplementation of probiotics and synbiotics enhancing calcium and phosphorus utilization, which improved the bone development of offspring pigs. Moreover, the maximum period of bone growth for pigs is in the post-natal 12 weeks [46]. Increased plasma ALT and AST activities are associated with improved amino acid metabolism [45]. The deamination of amino acids could produce AMM, which is involved in the synthesis and breakdown of animal proteins or amino acids [47]. The UN is the main nitrogenous product of protein and amino acid catabolism. Therefore, a decrease in the plasma UN concentration indicates an increase in the ability of amino acids in the blood to synthesize proteins. Furthermore, plasma AMM and UN concentrations could reflect protein metabolism and amino acid balance. Our results showed that sow-offspring diets supplemented with probiotics and synbiotics significantly decreased the plasma UN concentration at 95 d old and AMM concentration at 125 d old, probiotics supplementation significantly decreased the plasma AMM concentration at 95 d old, whereas synbiotics supplementation significantly increased the plasma ALT activity at 95 d old. These results indicate that sow-offspring diets supplemented with probiotics and synbiotics could improve the balance of amino acids and protein utilization.

The composition and proportion of muscle-fiber types are closely related to meat quality [48]. Among the four muscle-fiber types, *MyHCI* and *MyHCIIa* are positively correlated with high-quality meat, and *MyHCIIa* and *MyHCIIx* are the critical factors affecting the meat quality [49]. In our study, at 65 d old, the *MyHCI* and *MyHCIIa* expressions in the LDM were significantly upregulated by sow-offspring diets supplemented with probiotics and synbiotics, as well as in the PMM by probiotics supplementation, while significantly downregulated *MyHCIIb* expression; moreover, at 125 d old, *MyHCIIa* expression in the LDM and *MyHCIIx* expression in the PMM were significantly upregulated, and synbiotics supplementation significantly upregulated the *MyHCI* expression in the LDM. Thus, sow-offspring diets supplemented with probiotics and synbiotics could alter the composition of muscle-fiber types and then improve meat quality. Similarly, Tian et al. [50] also indicated that dietary *Lactobacillus reuteri* 1 supplementation could upregulate the *MyHCI* expression and downregulate *MyHCIIb* expression in the muscles of pigs. In addition, antibiotics also had positive effects on the muscle-fiber type to some extent.

Muscle regulatory factors play important roles in the proliferation and differentiation of muscle cells, formation and function of muscle fibers, and the muscle maturation [51]. *MyoD* and *Myf5* are involved in muscle formation, *MyoG* plays a pivotal role in the differentiation of myocytes into muscle fibers, and *Myf6* is essential for muscle maintenance [52]. In the present study, sow-offspring diets supplemented with probiotics and synbiotics significantly upregulated the *Myf6* expression in the LDM at 125 d old; probiotics supplementation significantly upregulated *Myf5* and *Myf6* expressions in the LDM at 65 d old, and *MyoD* and *Myf5* expressions in the LDM and *IGF1*, *Myf5*, *Myf6*, and *MyoG* expressions in the PMM at 95 d old; synbiotics supplementation significantly upregulated *MyoG* expression in the PMM and *Myf5* expression in the LDM and PMM at 65 d old. These results suggest that probiotics and synbiotics supplementation in sow-offspring diets could promote skeletal muscle growth in offspring pigs.

*MSTN* is a negative regulator of skeletal muscle mass [53] and is negatively correlated with muscle growth and development [54]. Sow-offspring diets supplemented with probiotics and synbiotics significantly downregulated the *MSTN* expression in the PMM at 65 d old in the present study. The results suggest that the expressions of genes that suppress muscle growth were downregulated by probiotics and synbiotics supplementation in sow-offspring diets, thereby promoting muscle growth and development in offspring pigs.

## 4. Materials and Methods

### 4.1. Animals, Diets, and Treatments

**Figure 2:**
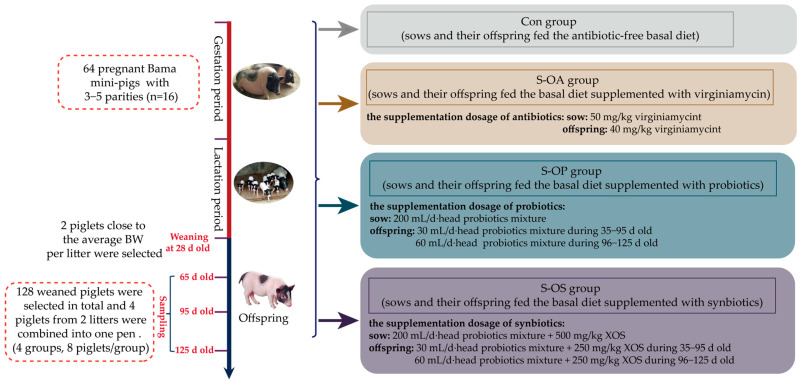
Schematic presentation of the experimental design.

During this experiment, animals were reared at the experimental base of the Institute of Subtropical Agriculture, Chinese Academy of Sciences, Changde, Hunan, China. Sixty-four pregnant Bama mini-pigs with 3–5 parities were randomly allocated into 4 groups (16 replicates per group), representing the control (basal diet without antibiotics), antibiotics (50 mg/kg pure virginiamycin + basal diet), probiotics (200 mL/d probiotics mixture each sow + basal diet), and synbiotics [200 mL/d probiotics mixture each sow + 500 mg/kg XOS + the basal diet] groups. These additives were added during the gestation and lactation period of the sow. During the gestation period, each sow was housed in an individual gestating crate (2.2 m × 0.6 m). The farrowing crates were used for sows and their respective piglets until weaning at 28 d old. The sows were fed with 0.8, 1.0, 1.2, 1.5, and 2.0 kg of pregnancy feed mix from days 1–15, 16–30, 31–75, 76–90, and 91–105 of pregnancy, respectively; fed with 1 kg of pregnancy feed mix a week before parturition and *ad libitum* after 3 days of parturition; and fed with 2.4 kg of a lactation feed mix until weaning. Experimental sows were fed twice daily at 8:00 am and 5:00 pm with their respective diets, and water was freely available at all times. To conduct the subsequent feeding trial, two piglets close to the average BW per litter were selected. A total of 128 piglets were transferred to the nursery house, and four piglets from two litters in the same group were fed in one pen, which had eight replicates (pens) in each treatment group. Offspring pigs were fed with a basal diet supplemented with the same additive, though different supplemental levels as their corresponding sows, and represented the Con group (sows and their offspring fed the antibiotic-free basal diet), S-OA group (sows and their offspring fed the basal diet supplemented with pure virginiamycin), S-OP group (sows and their offspring fed the basal diet supplemented with probiotics), and S-OS group (sows and their offspring fed the basal diet supplemented with synbiotics). In the offspring pig diets, the supplementation levels of pure virginiamycin were 40 mg/kg during 35–125 d old; the supplementation levels of probiotics were 30 mL/d·head, and those of synbiotics were 250 mg/kg XOS + 30 mL/d·head probiotics mixture during 35−95 d old; the supplementation levels of probiotics were 60 mL/d·head, and those of synbiotics were 250 mg/kg XOS + 60 mL/d·head probiotics mixture during 96−125 d old (Figure 2). The supplementing dose of probiotics and prebiotics was according to the manufacturer’s recommendations. The probiotics mixture was provided by Hunan Lifeng Biotechnology Co., Ltd. (Changsha, China), and contained *Lactobacillus plantarum* B90 (CGMCC1.12934) ≥ 1 × 10^8^ CFU/mL and *Saccharomyces cerevisiae* P11 (CGMCC2.3854) ≥ 0.2 × 10^8^ CFU/mL. The supplemented probiotics mixture was mixed with the feed before feeding the sows and offspring piglets, and XOS was added during feed production. The XOS (≥35%) was provided by Shandong Longlive Biotechnology Co., Ltd. (Dezhou, China), and contained xylobiose (55%), xylotriose (25%), xylotetraose (10%), xylopentose (5%), xylohexaose (3%), and xyloheptaose (2%), which met the feed additive of XOS recommended requirements (GB/T23747-2009). The basal diet composition and nutrient levels are shown in Tables S1 and S2. The composition, supplier, and feeding method of probiotics and synbiotics were consistent with those previously reported by Zhu et al. [15].

### 4.2. Determination of Growth Performance

The feed intake of offspring pigs per pen was recorded daily. At 65, 95, and 125 d old, the BW of all the offspring pigs was weighed. In addition, the ADG, ADFI, and F/G of offspring pigs were calculated from days 35 to 65, 66 to 95, and 96 to 125.

### 4.3. Sample Collection

The offspring pigs were fasted for 12 h and weighed at 65, 95, and 125 d old. Then eight pigs per group (one pig from each pen with an average BW per pen) at each time point of sampling were selected and euthanized using electrical stunning (120 V, 200 Hz) and exsanguination under commercial conditions. The heparinized tubes (10 mL) were used to collect blood samples via the precaval vein and then centrifuged at 4 °C and 3500× *g* for 10 min to obtain plasma. For further analysis, the plasma samples were immediately stored at −20 °C. After dissection, the head, legs, tail, and viscera of pigs were removed, and the carcass was split longitudinally to evaluate the carcass traits and meat quality. To analyze the routine nutrient composition, the LDM and PMM were separated and stored in sealed plastic bags at −20 °C. For analysis of the mRNA expression, LDM and PMM were also collected and stored at −80 °C after being frozen in liquid nitrogen.

### 4.4. Determination of Carcass Traits and Meat Quality

To determine carcass quality indicators, the left side of each carcass was dissected in accordance with the standard (GB8467-87, 1988) protocol. Fat percentage, bone percentage, muscle percentage, and the leaf-fat ratio of offspring pigs were calculated as previously reported by Zhu et al. [15], namely, tissue weight × 2 (kg)/BW (kg) × 100 and leaf-fat weight × 2 (g)/BW (kg). A Vernier caliper was used to measure backfat thickness between the sixth and seventh ribs and the width and height of the LDM cross section. The loin-eye muscle area was calculated by the width and height of the LDM cross section (width × height × 0.7).

According to the National Pork Producers Council [55], meat color scores and subjective marbling scores were determined for the LDM. A colorimeter (CR410; Konica Minolta Sensing, Inc., Tokyo, Japan) was used to determine the meat color (L*, a*, and b* values) of the LDM at 45 min postmortem on the surface. A portable pH meter (Russell CD700; Russell pH Limited, München, Germany) was used to determine the pH_45min_ and pH_24h_ postmortem. Drip loss was determined as previously reported by Honikel [56]. The cooking yield and shear force (N) were determined according to the methods described by previous studies [15,57]. Briefly, the LDM samples were placed in individual polyethylene vacuum bags and cooked for 30 min, and then the cooked samples were cooled to room temperature, dried with a paper towel, and reweighed to calculate the cooking yield (%) [(cooked weight/fresh weight) × 100]. The cooked samples were further trimmed parallel to the muscle fiber into strips with a diameter of 12.7 mm and a length of 20 mm, and then the strips were then used to determine shear force (N) using a texture analyzer (FTCTMS/PRO; FTC Corporation, Sterling, VA, USA) with a load cell of 15 kg and a crosshead speed of 200 mm/min.

### 4.5. Chemical Composition Analysis of Skeletal Muscle

After weighing, the skeletal muscles (including LDM and PMM) were minced and dried in a vacuum-freeze dryer (CHRIST RVC2-25 CDPIUS; Christ Company, Oster ode, Germany) for 72 h at 20 ± 5 Pa and −45 ± 5 °C to determine the chemical composition of the skeletal muscles.

Crude protein (CP) content (N × 6.25) was determined using the Kjeldahl method and following the standards provided by the Association of Official Analytical Chemists (AOAC) [58].

The hydrolytic amino acid content of the LDM and PMM was analyzed using an automatic amino acid analyzer (L-8900; Hitachi, Tokyo, Japan). The pretreatment method was as described in a previous study [15]. The content of TAA, EAA, NEAA, and FAA was calculated.

### 4.6. Analysis of Relative Expression of Target Genes by Quantitative RT-PCR

Following the manufacturer’s protocol, the total RNA was extracted from LDM and PMM samples using the AG RNAex Pro reagent (Accurate Biology, Changsha, China), and 1000 ng of extracted RNA were reverse transcribed to cDNA using the Evo M-MLV RT Kit (Accurate Biology) for quantitative PCR analysis. The cDNA was mixed with the 2 × SYBR^®^ Green Pro Taq HS Premix (Accurate Biology, Changsha, China) and amplified on the LightCycler^®^ 480II Real-Time PCR System (Roche, Basel, Switzerland). The reaction system and cycling conditions of RT-qPCR were as described previously by Zhu et al. [15]. Gene-specific primers are listed in Table S3. The relative expression of the target gene was calculated using the 2^−ΔΔCt^ method [59].

### 4.7. Determination of Plasma Parameters Associated with Nitrogen Metabolism

The activities of ALT, ALP, and AST, as well as the concentrations of ALB, AMM, TP, and UN, were measured using the Roche automatic biochemical analyzer (Cobas c311, F. Hoffmann-La Roche Ltd., Basel, Switzerland). In addition, an automatic amino acid analyzer (L8900; Hitachi, Tokyo, Japan) was used to analyze the plasma-free amino acids.

### 4.8. Statistical Analysis

The pen was considered the experimental unit for the growth performance data. Individual pigs were the experimental unit for other data. All the data were analyzed using one-way ANOVA of the SPSS (SPSS v. 25.0; SPSS Inc., Chicago, IL, USA) and Tukey’s post hoc test after the normal distribution of the data was confirmed by the Shapiro–Wilk test. The significant difference was set at *p* < 0.05 for all analyses. Results are presented as the means with their pooled standard error of the means (SEM).

## 5. Conclusions

In conclusion, probiotics supplementation in sow-offspring diets increased the feed intake and the growth of offspring pigs during 95–125 d old. Moreover, dietary probiotics supplementation improved muscle mass, fat deposition, and meat tenderness of offspring pigs. Furthermore, dietary probiotics and synbiotics supplementation improved meat quality by improving sensory indexes (including tenderness, water-holding capacity, and meat flavor); enhancing the balance of amino acids and protein utilization; promoting muscle growth and development and AA deposition in muscle; and altering muscle-fiber types composition of offspring pigs. The improvement effects on the growth performance and meat quality by dietary probiotics and synbiotics supplementation may be related to the regulation of these additives on the body’s metabolism and the growth and development of muscle. These discoveries provide a theoretical basis for the maternal-offspring integration regulation of meat quality by dietary probiotics and synbiotics supplementation.

## Figures and Tables

**Table 1:** Effects of probiotics and synbiotics supplementation in sow-offspring diets on growth performance of offspring pigs.

Items	Con Group	S-OA Group	S-OP Group	S-OS Group	SEM	*p*-Values
BW, kg						
35 d old	4.97	5.04	4.71	4.78	0.135	0.275
65 d old	9.37	9.25	9.49	8.96	0.168	0.162
95 d old	14.05	15.16	13.52	13.61	0.548	0.151
125 d old	22.67 ^b^	27.23 ^a^	28.29 ^a^	19.28 ^c^	0.912	<0.001
ADG, kg/d						
35–65 d old	0.15	0.15	0.15	0.14	0.006	0.435
66–95 d old	0.18	0.17	0.16	0.17	0.012	0.693
96–125 d old	0.27 ^b^	0.30 ^b^	0.37 ^a^	0.18 ^c^	0.023	<0.001
ADFI, kg/d						
35–65 d old	0.41	0.43	0.42	0.44	0.009	0.312
66–95 d old	0.64	0.65	0.69	0.59	0.025	0.081
96–125 d old	0.92 ^b^	1.29 ^a^	1.16 ^a^	0.71 ^c^	0.068	<0.001
F/G						
35–65 d old	3.02	2.93	2.99	3.29	0.160	0.410
66–95 d old	3.56	4.11	4.21	3.90	0.216	0.160
96–125 d old	3.32 ^b^	4.39 ^a^	2.95 ^b^	3.49 ^b^	0.211	0.002

Note: ^a–c^ Means within the same row not followed by the same letter differ significantly (*p* < 0.05). BW, body weight; ADG, average daily gain; ADFI, average daily feed intake; F/G, feed intake to body gain ratio; Con group: sow and offspring pigs fed with a basal diet; S-OA group: sow and offspring pigs fed with antibiotics; S-OP group: sow and offspring pigs fed with probiotics; S-OS group: sow and offspring pigs fed with synbiotics. The replicates of four groups during 35–65 d old were 8. The replicates of the Con, S-OA, S-OP, and S-OS groups during 66–95 d old were 8, 8, 8, and 7, respectively. The replicates of the Con, S-OA, S-OP, and S-OS groups during 96–125 d old were 8, 5, 6, and 6, respectively. The replicates per group at 65 d old were 8. The replicates of the Con, S-OA, S-OP, and S-OS groups at 95 d old were 8, 8, 8, and 7, respectively. The replicates of the Con, S-OA, S-OP, and S-OS groups at 125 d old were 8, 5, 6, and 6, respectively.

**Table 2:** Effects of probiotics and synbiotics supplementation in sow-offspring diets on carcass traits of offspring pigs.

Item	Con Group	S-OA Group	S-OP Group	S-OS Group	SEM	*p*-Values
Backfat thickness, mm						
65 d old	13.27 ^b^	14.77 ^a^	13.37 ^b^	13.12 ^b^	0.376	0.015
95 d old	18.79 ^a^	15.59 ^b^	16.65 ^b^	16.44 ^b^	0.537	0.002
125 d old	24.75 ^b^	27.37 ^ab^	30.98 ^a^	22.57 ^b^	1.434	0.003
Carcass weight, kg						
65 d old	5.00	4.60	5.01	4.70	0.141	0.108
95 d old	8.25	8.25	8.10	7.42	0.376	0.390
125 d old	13.83 ^b^	17.95 ^a^	17.20 ^a^	12.13 ^b^	0.981	0.001
Fat percentage, %						
65 d old	12.14 ^ab^	10.32 ^b^	13.43 ^a^	11.48 ^b^	0.540	0.003
95 d old	15.42 ^a^	12.55 ^b^	15.45 ^a^	13.83 ^ab^	0.503	0.001
125 d old	17.73 ^b^	18.85 ^b^	23.84 ^a^	18.28 ^b^	0.904	<0.001
Bone percentage, %						
65 d old	11.80 ^a^	11.14 ^b^	11.97 ^a^	11.18 ^b^	0.177	0.003
95 d old	10.13	10.40	10.32	10.91	0.243	0.180
125 d old	9.15 ^b^	8.57 ^b^	8.51 ^b^	10.04 ^a^	0.262	0.002
Muscle percentage, %						
65 d old	21.56 ^ab^	20.60 ^b^	22.14 ^a^	22.72 ^a^	0.428	0.011
95 d old	21.23 ^b^	22.37 ^b^	24.38 ^a^	21.66 ^b^	0.411	<0.001
125 d old	23.87 ^a^	23.24 ^ab^	23.10 ^ab^	22.36 ^b^	0.336	0.025
Leaf-fat ratio, g/kg						
65 d old	6.93 ^ab^	7.28 ^ab^	7.83 ^a^	5.98 ^b^	0.405	0.024
95 d old	11.41 ^a^	6.99 ^b^	13.00 ^a^	12.61 ^a^	0.785	<0.001
125 d old	16.77 ^b^	16.69 ^b^	24.54 ^a^	19.40 ^b^	1.254	0.001
Loin-eye muscle area, cm^2^						
65 d old	4.21 ^a^	3.39 ^b^	3.79 ^a^	3.85 ^a^	0.120	0.001
95 d old	4.33 ^c^	5.40 ^ab^	5.97 ^a^	4.73 ^bc^	0.302	0.003
125 d old	7.20 ^a^	6.57 ^a^	7.19 ^a^	5.65 ^b^	0.248	0.001

Note: ^aߝc^ Means within the same row not followed by the same letter differ significantly (*p* < 0.05). Con group: sow and offspring pigs fed with a basal diet; S-OA group: sow and offspring pigs fed with antibiotics; S-OP group: sow and offspring pigs fed with probiotics; S-OS group: sow and offspring pigs fed with synbiotics. The replicates per group at 65 d old were 8. The replicates of the Con, S-OA, S-OP, and S-OS groups at 95 d old were 8, 8, 8, and 7, respectively. The replicates of the Con, S-OA, S-OP, and S-OS groups at 125 d old were 8, 5, 6, and 6, respectively.

**Table 3:** Effects of probiotics and synbiotics supplementation in sow-offspring diets on meat quality of offspring pigs.

Item	Con Group	S-OA Group	S-OP Group	S-OS Group	SEM	*p*-Values
Marbling score						
65 d old	1.75	1.75	1.63	1.50	0.175	0.708
95 d old	1.88	1.75	2.00	1.86	0.287	0.941
125 d old	1.50	1.60	1.83	1.33	0.204	0.398
Meat color score						
65 d old	3.13	3.25	2.88	2.50	0.308	0.345
95 d old	2.50	2.38	2.25	2.29	0.181	0.763
125 d old	2.75	2.80	2.83	2.83	0.279	0.995
a* value						
65 d old	19.58	19.68	19.82	18.64	0.534	0.404
95 d old	18.44 ^bc^	19.56 ^a^	19.16 ^ab^	18.03 ^c^	0.255	0.001
125 d old	18.14	17.03	18.68	18.20	0.392	0.068
b* value						
65 d old	6.87	6.86	6.67	6.98	0.163	0.623
95 d old	6.72	6.36	6.67	6.39	0.172	0.336
125 d old	7.07 ^b^	6.28 ^c^	7.29 ^b^	8.28 ^a^	0.17	<0.001
L* value						
65 d old	51.01 ^a^	49.28 ^b^	47.35 ^b^	48.78 ^b^	0.577	0.001
95 d old	50.16 ^a^	47.76 ^b^	47.78 ^b^	49.49 ^ab^	0.662	0.030
125 d old	50.94	50.42	50.46	50.58	1.571	0.994
pH_45min_						
65 d old	6.55	6.49	6.45	6.51	0.058	0.697
95 d old	6.54 ^ab^	6.66 ^a^	6.41 ^b^	6.55 ^ab^	0.043	0.004
125 d old	6.49 ^b^	6.54 ^b^	6.66 ^ab^	6.74 ^a^	0.053	0.008
pH_24h_						
65 d old	5.46 ^b^	5.50 ^ab^	5.62 ^a^	5.51 ^ab^	0.037	0.036
95 d old	5.46 ^a^	5.43 ^b^	5.49 ^a^	5.48 ^a^	0.008	<0.001
125 d old	5.46 ^b^	5.52 ^a^	5.47 ^b^	5.46 ^b^	0.015	0.021
Drip loss, %						
65 d old	6.32 ^a^	3.46 ^c^	3.21 ^c^	4.43 ^b^	0.260	<0.001
95 d old	3.42 ^a^	2.72 ^ab^	2.24 ^b^	2.48 ^b^	0.265	0.023
125 d old	4.82 ^a^	2.11 ^b^	1.96 ^b^	3.08 ^b^	0.440	<0.001
Cooking yield, %						
65 d old	63.01 ^b^	61.73 ^b^	65.12 ^ab^	67.60 ^a^	1.077	0.004
95 d old	70.92 ^b^	73.80 ^a^	69.94 ^b^	70.29 ^b^	0.869	0.015
125 d old	68.57	67.64	69.87	67.56	0.609	0.055
Shear force, N						
65 d old	61.50 ^b^	60.79 ^b^	55.62 ^b^	78.16 ^a^	2.219	<0.001
95 d old	73.25	65.15	71.84	68.55	2.894	0.210
125 d old	91.94 ^a^	81.61 ^b^	73.89 ^b^	73.29 ^b^	3.304	0.001

Note: ^a–c^ Means within the same row not followed by the same letter differ significantly (*p* < 0.05). a* value, redness value; b* value, yellowness value; L* value, lightness value. Con group: sow and offspring pigs fed with a basal diet; S-OA group: sow and offspring pigs fed with antibiotics; S-OP group: sow and offspring pigs fed with probiotics; S-OS group: sow and offspring pigs fed with synbiotics. The replicates per group at 65 d old were 8. The replicates of the Con, S-OA, S-OP, and S-OS groups at 95 d old were 8, 8, 8, and 7, respectively. The replicates of the Con, S-OA, S-OP, and S-OS groups at 125 d old were 8, 5, 6, and 6, respectively.

**Table 4:** Effects of probiotics and synbiotics supplementation in sow-offspring diets on crude protein and amino acids content in *longissimus dorsi* muscle of offspring pigs (g/100 g fresh muscle).

Item	Con Group	S-OA Group	S-OP Group	S-OS Group	SEM	*p*-Values
CP						
65 d old	23.39	23.59	23.84	22.94	0.390	0.435
95 d old	22.12	22.97	22.64	22.69	0.397	0.492
125 d old	22.21	22.39	22.55	22.25	0.376	0.910
Ala						
65 d old	1.20 ^ab^	1.28 ^a^	1.17 ^b^	1.16 ^b^	0.030	0.034
95 d old	1.06 ^b^	1.36 ^a^	1.28 ^a^	1.27 ^a^	0.043	<0.001
125 d old	1.17 ^b^	1.23 ^ab^	1.27 ^a^	1.28 ^a^	0.029	0.038
Arg						
65 d old	1.34 ^ab^	1.40 ^a^	1.32 ^ab^	1.26 ^b^	0.032	0.031
95 d old	1.21	1.27	1.22	1.26	0.038	0.635
125 d old	1.34 ^b^	1.43 ^ab^	1.46 ^a^	1.42 ^ab^	0.027	0.027
Asp						
65 d old	1.80 ^a^	1.85 ^a^	1.70 ^ab^	1.57 ^b^	0.053	0.005
95 d old	1.51 ^b^	1.83 ^a^	1.79 ^a^	1.77 ^a^	0.059	0.002
125 d old	1.76 ^b^	1.74 ^b^	1.90 ^a^	1.70 ^b^	0.044	0.020
Glu						
65 d old	2.63	2.71	2.54	2.55	0.067	0.269
95 d old	2.63	2.67	2.57	2.59	0.090	0.879
125 d old	2.84 ^b^	3.03 ^a^	3.10 ^a^	2.73 ^b^	0.076	0.011
Gly						
65 d old	1.09 ^a^	1.14 ^a^	1.02 ^a^	0.95 ^b^	0.035	0.007
95 d old	0.95 ^b^	1.09 ^a^	1.01 ^ab^	1.05 ^ab^	0.033	0.037
125 d old	0.98	0.98	0.97	1.03	0.058	0.870
His						
65 d old	0.80	0.82	0.80	0.77	0.043	0.854
95 d old	0.94 ^a^	0.83 ^b^	0.78 ^b^	0.77 ^b^	0.027	0.001
125 d old	0.89 ^b^	1.06 ^a^	1.02 ^ab^	1.09 ^a^	0.055	0.048
Ile						
65 d old	0.90 ^ab^	0.93 ^a^	0.84 ^b^	0.83 ^b^	0.026	0.042
95 d old	0.82	0.89	0.89	0.92	0.030	0.118
125 d old	0.99 ^ab^	1.01 ^ab^	1.07 ^a^	0.97 ^b^	0.023	0.049
Leu						
65 d old	1.74 ^a^	1.81 ^a^	1.66 ^a^	1.50 ^b^	0.052	0.001
95 d old	1.43 ^b^	1.73 ^a^	1.71 ^a^	1.70 ^a^	0.055	0.001
125 d old	1.67	1.68	1.82	1.69	0.047	0.113
Lys						
65 d old	1.77 ^ab^	1.84 ^a^	1.68 ^ab^	1.63 ^b^	0.047	0.017
95 d old	1.59	1.70	1.67	1.67	0.056	0.550
125 d old	1.89 ^ab^	1.97 ^ab^	2.04 ^a^	1.83 ^b^	0.049	0.035
Met						
65 d old	0.53	0.54	0.51	0.50	0.017	0.351
95 d old	0.54	0.50	0.49	0.51	0.016	0.108
125 d old	0.62	0.67	0.67	0.63	0.019	0.180
Phe						
65 d old	0.87	0.89	0.83	0.80	0.025	0.073
95 d old	0.86	0.78	0.79	0.82	0.028	0.180
125 d old	0.90	0.98	0.99	0.96	0.032	0.175
Pro						
65 d old	0.94 ^b^	1.04 ^ab^	0.96 ^b^	1.08 ^a^	0.037	0.031
95 d old	1.17 ^a^	0.81 ^b^	0.76 ^b^	0.90 ^b^	0.061	<0.001
125 d old	1.04 ^b^	1.25 ^ab^	1.09 ^b^	1.35 ^a^	0.068	0.013
Ser						
65 d old	0.69	0.72	0.67	0.66	0.016	0.064
95 d old	0.71	0.67	0.66	0.67	0.025	0.620
125 d old	0.75 ^b^	0.83 ^a^	0.82 ^ab^	0.76 ^b^	0.019	0.010
Tyr						
65 d old	0.71	0.74	0.68	0.65	0.023	0.053
95 d old	0.63	0.54	0.57	0.62	0.040	0.348
125 d old	0.75 ^b^	0.91 ^a^	0.85 ^ab^	0.82 ^ab^	0.035	0.024
Thr						
65 d old	0.93 ^ab^	0.99 ^a^	0.90 ^b^	0.86 ^b^	0.023	0.005
95 d old	0.84	0.88	0.88	0.91	0.032	0.483
125 d old	0.96 ^b^	1.05 ^a^	1.05 ^a^	0.99 ^ab^	0.025	0.024
Val						
65 d old	0.97 ^ab^	1.00 ^a^	0.92 ^ab^	0.89 ^b^	0.024	0.011
95 d old	0.96	0.97	0.93	0.93	0.029	0.794
125 d old	1.08	1.12	1.16	1.06	0.031	0.122
EAA						
65 d old	9.83 ^ab^	10.24 ^a^	9.49 ^ab^	9.04 ^b^	0.255	0.018
95 d old	9.17	9.54	9.37	9.50	0.287	0.792
125 d old	10.35	10.97	11.28	10.65	0.254	0.067
NEAA						
65 d old	9.08	9.48	8.81	8.63	0.217	0.055
95 d old	8.56	8.96	8.64	8.88	0.279	0.700
125 d old	9.29	9.97	10.00	9.67	0.200	0.052
FAA						
65 d old	8.06 ^ab^	8.38 ^a^	7.77 ^ab^	7.50 ^b^	0.196	0.022
95 d old	7.39	8.21	7.87	7.94	0.231	0.106
125 d old	8.09	8.40	8.71	8.16	0.170	0.063
TAA						
65 d old	18.90 ^ab^	19.71 ^a^	18.30 ^ab^	17.67 ^b^	0.461	0.027
95 d old	17.25	18.51	18.01	18.37	0.533	0.349
125 d old	19.64 ^b^	20.94 ^ab^	21.28 ^a^	20.32 ^ab^	0.413	0.036

Note: ^a,b^ Means within the same row not followed by the same letter differ significantly (*p* < 0.05). CP, crude protein; EAA, essential amino acids, including arginine (Arg), histidine (His), isoleucine (Ile), leucine (Leu), lysine (Lys), methionine (Met), phenylalanine (Phe), threonine (Thr), and valine (Val); NEAA, nonessential amino acids, including alanine (Ala), aspartic acid (Asp), glutamic acid (Glu), glycine (Gly), proline (Pro), serine (Ser), and tyrosine (Tyr); FAA, flavor amino acids, including Asp, Glu, Gly, Ala, and Arg. The content of Glu includes glutamine content. The content of Asp includes asparagine content. Con group: sow and offspring pigs fed with a basal diet; S-OA group: sow and offspring pigs fed with antibiotics; S-OP group: sow and offspring pigs fed with probiotics; S-OS group: sow and offspring pigs fed with synbiotics. The replicates of four groups at 65 d old were 8. The replicates of the Con, S-OA, S-OP, and S-OS groups at 95 d old were 8, 8, 8, and 7, respectively. The replicates of the Con, S-OA, S-OP, and S-OS groups at 125 d old were 8, 5, 6, and 6, respectively.

**Table 5:** Effects of probiotics and synbiotics supplementation in sow-offspring diets on crude protein and amino acids content in the *psoas major* muscle of offspring pigs (g/100 g fresh muscle).

Item	Con Group	S-OA Group	S-OP Group	S-OS Group	SEM	*p*-Values
CP						
65 d old	23.49 ^a^	24.62 ^a^	23.69 ^a^	22.33 ^b^	0.366	0.002
95 d old	22.46	21.60	22.22	21.88	0.314	0.237
125 d old	21.17	21.24	21.16	21.57	0.258	0.648
Ala						
65 d old	1.22 ^ab^	1.26 ^a^	1.08 ^b^	1.12 ^b^	0.045	0.026
95 d old	1.23 ^a^	1.28 ^a^	1.10 ^b^	1.08 ^b^	0.040	0.003
125 d old	1.13	1.14	1.23	1.23	0.043	0.199
Arg						
65 d old	1.33 ^b^	1.50 ^a^	1.39 ^ab^	1.40 ^ab^	0.035	0.020
95 d old	1.28	1.31	1.25	1.23	0.043	0.615
125 d old	1.29	1.25	1.37	1.41	0.042	0.057
Asp						
65 d old	1.95 ^a^	1.94 ^a^	1.78 ^ab^	1.70 ^b^	0.056	0.008
95 d old	1.69	1.77	1.65	1.62	0.064	0.401
125 d old	1.74	1.63	1.77	1.75	0.071	0.574
Glu						
65 d old	2.91 ^b^	3.27 ^a^	2.98 ^b^	2.84 ^b^	0.081	0.005
95 d old	2.79	2.87	2.67	2.67	0.085	0.264
125 d old	2.95	2.96	3.10	3.26	0.099	0.117
Gly						
65 d old	0.97 ^b^	1.07 ^a^	0.95 ^b^	0.90 ^b^	0.026	0.001
95 d old	0.85	0.94	0.83	0.86	0.043	0.276
125 d old	0.85	0.84	0.92	0.92	0.040	0.353
His						
65 d old	0.76	0.76	0.78	0.75	0.015	0.478
95 d old	0.86 ^a^	0.79 ^ab^	0.76 ^ab^	0.68 ^b^	0.040	0.025
125 d old	0.74 ^b^	0.92 ^a^	0.88 ^a^	0.84 ^ab^	0.036	0.008
Ile						
65 d old	0.94	1.03	0.94	0.94	0.028	0.068
95 d old	0.93	0.94	0.92	0.90	0.030	0.786
125 d old	0.94	0.88	0.98	1.01	0.038	0.148
Leu						
65 d old	1.73	1.75	1.63	1.60	0.044	0.053
95 d old	1.71	1.70	1.60	1.53	0.056	0.098
125 d old	1.59	1.57	1.67	1.65	0.061	0.656
Lys						
65 d old	2.00 ^a^	1.99 ^a^	1.85 ^ab^	1.78 ^b^	0.047	0.006
95 d old	1.66	1.76	1.72	1.73	0.059	0.700
125 d old	1.81	1.72	1.86	1.97	0.083	0.275
Met						
65 d old	0.56	0.62	0.57	0.57	0.016	0.086
95 d old	0.57	0.54	0.58	0.55	0.018	0.427
125 d old	0.55	0.59	0.63	0.62	0.028	0.244
Phe						
65 d old	0.82 ^b^	0.97 ^a^	0.89 ^a^	0.91 ^a^	0.023	0.001
95 d old	0.88	0.85	0.83	0.81	0.023	0.165
125 d old	0.85	1.00	1.00	1.01	0.048	0.051
Pro						
65 d old	0.76 ^c^	1.14 ^a^	0.98 ^b^	1.19 ^a^	0.045	<0.001
95 d old	1.03	0.99	0.90	0.85	0.054	0.114
125 d old	0.89 ^b^	0.98 ^b^	1.17 ^a^	1.20 ^a^	0.038	<0.001
Ser						
65 d old	0.78 ^b^	0.86 ^a^	0.79 ^b^	0.75 ^b^	0.019	0.003
95 d old	0.75	0.75	0.71	0.69	0.019	0.142
125 d old	0.74	0.81	0.80	0.82	0.028	0.142
Tyr						
65 d old	0.72 ^b^	0.81 ^ab^	0.76 ^ab^	0.84 ^a^	0.027	0.018
95 d old	0.65	0.64	0.72	0.67	0.026	0.152
125 d old	0.70 ^c^	0.22 ^d^	0.77 ^b^	0.85 ^a^	0.019	<0.001
Thr						
65 d old	0.97	1.05	0.93	0.97	0.029	0.061
95 d old	0.97 ^a^	0.95 ^a^	0.90 ^ab^	0.87 ^b^	0.026	0.046
125 d old	0.91 ^b^	0.90 ^b^	0.99 ^ab^	1.04 ^a^	0.029	0.009
Val						
65 d old	1.01 ^b^	1.10 ^a^	1.02 ^b^	0.99 ^b^	0.026	0.027
95 d old	0.96	0.97	0.97	0.96	0.028	0.981
125 d old	1.00	1.01	1.08	1.12	0.041	0.172
EAA						
65 d old	10.12	10.77	9.96	9.91	0.241	0.064
95 d old	9.71	9.80	9.52	9.24	0.264	0.475
125 d old	9.68	9.85	10.46	10.65	0.380	0.217
NEAA						
65 d old	9.39 ^b^	10.35 ^a^	9.31 ^b^	9.34 ^b^	0.238	0.011
95 d old	8.85	9.24	8.59	8.44	0.271	0.194
125 d old	9.00 ^bc^	8.68 ^c^	9.75 ^ab^	10.04 ^a^	0.286	0.012
FAA						
65 d old	8.39 ^b^	9.04 ^a^	8.08 ^b^	7.96 ^b^	0.218	0.007
95 d old	7.81	8.17	7.50	7.45	0.260	0.206
125 d old	7.96	7.82	8.38	8.57	0.281	0.239
TAA						
65 d old	19.51 ^b^	21.12 ^a^	19.27 ^b^	19.25 ^b^	0.473	0.026
95 d old	18.55	19.05	18.10	17.68	0.527	0.321
125 d old	18.68	18.52	20.21	20.69	0.660	0.071

Note: ^a–d^ Means within the same row not followed by the same letter differ significantly (*p* < 0.05). CP, crude protein; EAA, essential amino acids, including arginine (Arg), histidine (His), isoleucine (Ile), leucine (Leu), lysine (Lys), methionine (Met), phenylalanine (Phe), threonine (Thr), and valine (Val); NEAA, nonessential amino acids, including alanine (Ala), aspartic acid (Asp), glutamic acid (Glu), glycine (Gly), proline (Pro), serine (Ser), and tyrosine (Tyr); FAA, flavor amino acids, including Asp, Glu, Gly, Ala, and Arg. The content of Glu includes glutamine content. The content of Asp includes asparagine content. Con group: sow and offspring pigs fed with a basal diet; S-OA group: sow and offspring pigs fed with antibiotics; S-OP group: sow and offspring pigs fed with probiotics; S-OS group: sow and offspring pigs fed with synbiotics. The replicates per group at 65 d old were 8. The replicates of the Con, S-OA, S-OP, and S-OS groups at 95 d old were 8, 8, 8, and 7, respectively. The replicates of the Con, S-OA, S-OP, and S-OS groups at 125 d old were 8, 5, 6, and 6, respectively.

**Table 6:** Effects of probiotics and synbiotics supplementation in sow-offspring diets on plasma-free amino acids concentrations of offspring pigs (nmoL/mL).

Item	Con Group	S-OA Group	S-OP Group	S-OS Group	SEM	*p*-Values
1Mehis						
65 d old	0.85	0.95	0.50	0.90	0.147	0.157
95 d old	6.13 ^b^	8.96 ^a^	5.06 ^b^	6.70 ^b^	0.626	0.001
125 d old	0.93	0.89	0.44	1.16	0.201	0.095
3Mehis						
65 d old	12.38	11.64	11.69	12.13	0.766	0.885
95 d old	14.09	13.74	12.71	15.27	0.730	0.123
125 d old	13.92	15.41	17.17	16.37	1.082	0.146
Ala						
65 d old	419.55 ^a^	445.57 ^a^	323.57 ^b^	391.40 ^ab^	24.723	0.011
95 d old	358.50 ^a^	252.02 ^b^	296.22 ^ab^	347.86 ^a^	18.048	0.001
125 d old	312.99 ^b^	393.20 ^a^	301.66 ^b^	284.56 ^b^	20.815	0.009
Ans						
65 d old	1.05	1.04	0.98	0.88	0.105	0.682
95 d old	0.48 ^a^	0.46 ^a^	0.51 ^a^	0.36 ^b^	0.028	0.005
125 d old	22.53 ^c^	55.86 ^a^	35.01 ^b^	21.56 ^c^	2.124	<0.001
Arg						
65 d old	70.36	79.78	83.70	88.64	4.783	0.070
95 d old	89.22 ^ab^	67.25 ^b^	69.73 ^b^	102.12 ^a^	6.953	0.004
125 d old	84.84 ^b^	105.52 ^a^	85.15 ^b^	88.29 ^b^	3.540	0.001
Asp						
65 d old	7.88	9.41	8.84	7.39	1.256	0.665
95 d old	9.86	10.99	11.55	9.53	0.748	0.211
125 d old	10.85 ^a^	12.44 ^a^	6.99 ^b^	10.34 ^a^	0.679	<0.001
Car						
65 d old	10.51	10.61	10.72	7.03	1.152	0.087
95 d old	10.93 ^a^	8.97 ^a^	3.30 ^b^	8.24 ^a^	0.985	<0.001
125 d old	18.25	17.06	13.97	15.10	1.300	0.083
Cit						
65 d old	25.65 ^c^	30.35 ^b^	23.54 ^c^	34.81 ^a^	1.272	<0.001
95 d old	40.77 ^a^	31.80 ^b^	30.24 ^b^	34.47 ^b^	1.829	0.002
125 d old	38.50	34.22	40.66	34.89	2.474	0.229
Cysthi						
65 d old	13.04 ^b^	7.55 ^c^	13.52 ^b^	18.79 ^a^	0.927	<0.001
95 d old	6.05 ^b^	6.69 ^b^	7.17 ^ab^	8.37 ^a^	0.437	0.006
125 d old	9.97 ^b^	9.13 ^b^	10.88 ^ab^	13.89 ^a^	1.027	0.036
Cys						
65 d old	9.56	11.19	8.58	12.69	1.062	0.052
95 d old	94.43	93.56	59.16	70.89	14.719	0.264
125 d old	12.55	11.11	7.17	12.87	1.872	0.115
EOHNH_2_						
65 d old	0.27	0.31	0.25	0.28	0.031	0.558
95 d old	24.52 ^b^	36.69 ^a^	0.46 ^c^	0.27 ^c^	2.086	<0.001
125 d old	5.09 ^a^	5.31 ^a^	5.90 ^a^	2.95 ^b^	0.522	0.006
Glu						
65 d old	186.00	211.73	197.39	183.61	11.824	0.334
95 d old	207.52	175.66	213.62	215.32	11.319	0.066
125 d old	155.75 ^a^	133.41 ^b^	159.41 ^a^	134.23 ^b^	4.571	<0.001
Gly						
65 d old	525.00 ^a^	441.15 ^b^	417.84 ^b^	517.45 ^a^	22.405	0.003
95 d old	470.34	472.11	484.09	560.27	29.847	0.129
125 d old	566.31 ^b^	744.00 ^a^	517.49 ^b^	582.66 ^b^	20.194	<0.001
His						
65 d old	36.13 ^bc^	38.06 ^ab^	39.58 ^a^	35.05 ^c^	0.834	0.003
95 d old	43.72 ^a^	38.14 ^b^	37.98 ^b^	40.04 ^ab^	1.406	0.026
125 d old	44.72 ^a^	47.79 ^a^	39.29 ^b^	44.59 ^a^	1.074	<0.001
Hylys						
65 d old	1.08 ^b^	0.99 ^b^	1.77 ^a^	1.00 ^b^	0.144	0.001
95 d old	0.55 ^b^	0.21 ^b^	0.31 ^b^	17.46 ^a^	0.545	<0.001
125 d old	20.27	17.00	16.29	13.96	2.598	0.404
Hypro						
65 d old	80.45 ^a^	63.26 ^b^	67.49 ^b^	63.80 ^b^	3.157	0.002
95 d old	37.70	27.34	31.12	40.87	3.707	0.062
125 d old	67.51	73.25	61.61	56.72	4.385	0.098
Ile						
65 d old	100.01 ^a^	89.39 ^b^	85.87 ^b^	106.56 ^a^	3.376	0.001
95 d old	90.11 ^a^	72.10 ^b^	68.44 ^b^	75.76 ^b^	3.811	0.002
125 d old	86.29 ^b^	98.37 ^ab^	95.59 ^ab^	101.40 ^a^	3.731	0.042
Leu						
65 d old	164.89 ^a^	150.55 ^ab^	141.18 ^b^	157.55 ^ab^	4.946	0.015
95 d old	140.61 ^a^	110.86 ^b^	108.49 ^b^	101.71 ^b^	6.546	0.001
125 d old	142.49 ^c^	164.70 ^b^	145.71 ^c^	188.21 ^a^	5.000	<0.001
Lys						
65 d old	143.17	132.49	136.97	143.94	6.986	0.619
95 d old	125.61	124.84	107.98	137.62	9.034	0.167
125 d old	135.46 ^bc^	152.98 ^a^	124.49 ^c^	146.49 ^ab^	4.722	0.001
Met						
65 d old	15.68	15.23	14.20	14.48	0.603	0.306
95 d old	12.76	10.83	10.76	11.79	0.641	0.113
125 d old	12.73 ^c^	16.16 ^a^	14.10 ^b^	12.84 ^c^	0.305	<0.001
Orn						
65 d old	51.91	55.78	59.34	56.87	3.443	0.500
95 d old	56.21 ^a^	40.61 ^b^	53.07 ^a^	64.72 ^a^	4.032	0.002
125 d old	68.98 ^a^	55.47 ^b^	43.97 ^b^	56.39 ^b^	3.736	<0.001
Phe						
65 d old	81.15	85.83	78.38	79.57	3.381	0.436
95 d old	81.64	79.95	73.05	83.18	2.810	0.077
125 d old	90.72 ^ab^	92.96 ^a^	85.38 ^b^	88.08 ^ab^	1.540	0.009
Pro						
65 d old	173.12 ^b^	214.35 ^a^	163.76 ^b^	188.85 ^b^	7.593	<0.001
95 d old	157.10	143.26	154.65	148.96	8.298	0.650
125 d old	183.79 ^b^	224.84 ^a^	162.65 ^b^	160.26 ^b^	11.179	0.002
Sar						
65 d old	8.53 ^a^	3.72 ^b^	4.13 ^b^	6.10 ^ab^	0.875	0.002
95 d old	0.41 ^c^	0.66 ^c^	6.67 ^a^	2.08 ^b^	0.381	<.0.001
125 d old	2.25	2.38	2.28	2.28	0.115	0.855
Ser						
65 d old	75.51	87.29	82.69	80.79	3.228	0.101
95 d old	81.57	75.69	78.11	75.35	2.589	0.318
125 d old	81.30 ^b^	97.57 ^a^	69.65 ^c^	73.61 ^c^	1.802	<0.001
Tau						
65 d old	133.56 ^a^	108.96 ^bc^	100.33 ^c^	121.25 ^ab^	5.264	0.001
95 d old	138.40 ^a^	116.37 ^b^	116.01 ^b^	116.55 ^b^	5.698	0.022
125 d old	137.88 ^b^	153.35 ^a^	133.68 ^b^	128.73 ^b^	5.074	0.021
Thr						
65 d old	116.70 ^b^	151.48 ^a^	108.88 ^b^	117.32 ^b^	7.145	0.001
95 d old	105.91	107.08	113.80	114.86	8.913	0.852
125 d old	119.87 ^b^	147.49 ^a^	111.62 ^b^	113.26 ^b^	4.004	<0.001
Tyr						
65 d old	50.03 ^a^	47.53 ^a^	24.34 ^b^	47.37 ^a^	4.762	0.002
95 d old	44.93	53.06	48.90	52.60	2.562	0.112
125 d old	62.07 ^b^	67.95 ^a^	60.31 ^b^	60.48 ^b^	1.55	0.008
Val						
65 d old	249.16 ^ab^	222.48 ^b^	220.34 ^b^	264.19 ^a^	12.257	0.047
95 d old	213.22	183.85	205.35	209.81	8.517	0.089
125 d old	237.37 ^b^	283.85 ^a^	257.01 ^ab^	284.97 ^a^	13.022	0.044
α-AAA						
65 d old	65.81	56.64	67.93	57.40	4.768	0.247
95 d old	60.05 ^a^	45.61 ^b^	47.63 ^b^	45.07 ^b^	2.733	0.002
125 d old	55.04 ^ab^	49.07 ^ab^	57.68 ^a^	44.23 ^b^	3.369	0.049
α-ABA						
65 d old	15.47 ^b^	24.10 ^a^	15.68 ^b^	18.36 ^ab^	2.277	0.042
95 d old	12.16 ^a^	4.47 ^b^	0.66 ^c^	4.87 ^b^	1.147	<0.001
125 d old	2.68 ^c^	3.45 ^b^	3.70 ^ab^	4.06 ^a^	0.131	<0.001
β-Ala						
65 d old	8.33	5.87	6.00	7.30	0.763	0.096
95 d old	8.88 ^a^	3.91 ^c^	2.70 ^c^	6.33 ^b^	0.555	<0.001
125 d old	6.91 ^b^	8.60 ^a^	6.97 ^b^	7.85 ^ab^	0.273	<0.001
β-AiBA						
65 d old	0.52 ^b^	1.43 ^a^	0.33 ^b^	1.35 ^a^	0.084	<0.001
95 d old	0.66 ^c^	2.55 ^a^	1.72 ^b^	1.73 ^b^	0.196	<0.001
125 d old	0.61 ^b^	1.05 ^a^	0.59 ^b^	0.37 ^c^	0.054	<0.001
γ-ABA						
65 d old	0.25 ^b^	0.21 ^b^	0.16 ^b^	0.59 ^a^	0.041	<0.001
95 d old	0.67 ^a^	0.23 ^b^	0.18 ^b^	0.16 ^b^	0.060	<0.001
125 d old	1.81	1.89	1.66	1.64	0.115	0.374

Note: ^a–c^ Means within the same row not followed by the same letter differ significantly (*p* < 0.05). 1Mehis, 1-methyl-histidine; 3Mehis, 3-methyl-histidine; Ala, alanine; Ans, anserine; Arg, arginine; Asp, aspartic acid; Car, carnosine; Cit, citrulline; Cysthi, cystathionine; Cys, cysteine; EOHNH_2_, ethanolamine; Glu, glutamic acid; Gly, glycine; His, histidine; Hylys, hydroxy-lysine; Hypro, hydroxy-proline; Ile, isoleucine; Leu, leucine; Lys, lysine; Met, methionine; Orn, ornithine; Phe, phenylalanine; Pro, proline; Sar, sarcosine; Ser, serine; Tau, taurine; Thr, threonine; Tyr, tyrosine; Val, valine; α-AAA, α-aminoadipic acid; α-ABA, α-amino-n-butyric acid; β-Ala, β-alanine; β-AiBA, β-aminoisobutyric acid; γ-ABA, γ-amino-n-butyric acid. Con group: sow and offspring pigs fed with a basal diet; S-OA group: sow and offspring pigs fed with antibiotics; S-OP group: sow and offspring pigs fed with probiotics; S-OS group: sow and offspring pigs fed with synbiotics. The replicates per group at 65 d old were 8. The replicates of the Con, S-OA, S-OP, and S-OS groups at 95 d old were 8, 8, 8, and 7, respectively. The replicates of the Con, S-OA, S-OP, and S-OS groups at 125 d old were 8, 5, 6, and 6, respectively.

**Table 7:** Effects of probiotics and synbiotics supplementation in sow-offspring diets on plasma biochemical parameters of offspring pigs.

Items	Con Group	S-OA Group	S-OP Group	S-OS Group	SEM	*p*-Values
ALT, U/L						
65 d old	58.79 ^a^	48.30 ^b^	54.03 ^ab^	61.18 ^a^	2.196	0.001
95 d old	54.74 ^b^	48.62 ^b^	53.06 ^b^	65.55 ^a^	2.048	<0.001
125 d old	51.77	48.75	55.33	50.90	4.307	0.775
ALP, U/L						
65 d old	148.88 ^b^	128.00 ^b^	184.43 ^a^	189.80 ^a^	7.237	<0.001
95 d old	123.63 ^ab^	146.33 ^a^	115.40 ^ab^	102.00 ^b^	10.164	0.035
125 d old	162.88	153.50	133.20	166.50	14.843	0.401
AST, U/L						
65 d old	59.75	78.83	70.71	83.80	7.388	0.134
95 d old	73.00 ^ab^	89.67 ^a^	51.83 ^b^	51.20 ^b^	6.731	0.001
125 d old	65.17	47.25	45.80	53.40	5.787	0.075
ALB, g/L						
65 d old	41.23	41.34	44.01	42.68	0.868	0.101
95 d old	40.45 ^ab^	37.98 ^b^	40.20 ^ab^	42.08 ^a^	0.717	0.005
125 d old	43.86	44.43	43.66	42.10	0.811	0.262
AMM, μmol/L						
65 d old	167.93 ^b^	269.70 ^a^	275.55 ^a^	188.83 ^b^	11.777	<0.001
95 d old	265.37 ^a^	239.43 ^ab^	169.36 ^b^	221.96 ^ab^	20.196	0.015
125 d old	332.90 ^a^	143.90 ^b^	176.60 ^b^	189.38 ^b^	19.774	<0.001
TP, g/L						
65 d old	65.00 ^c^	73.62 ^a^	69.39 ^b^	66.23 ^bc^	1.125	<0.001
95 d old	73.68 ^a^	73.32 ^a^	68.96 ^b^	73.52 ^a^	0.850	0.001
125 d old	72.76 ^ab^	69.28 ^b^	69.58 ^b^	75.92 ^a^	1.110	0.001
UN, mmol/L						
65 d old	3.10 ^b^	3.91 ^ab^	4.24 ^a^	3.38 ^ab^	0.292	0.042
95 d old	3.56 ^a^	3.12 ^b^	2.47 ^c^	2.96 ^b^	0.141	<0.001
125 d old	3.47 ^ab^	3.10 ^b^	4.08 ^a^	3.00 ^b^	0.214	0.009

Note: ^a–c^ Means within the same row not followed by the same letter differ significantly (*p* < 0.05). ALT, alanine aminotransferase; ALP, alkaline phosphatase; AST, aspartate aminotransferase; ALB, albumin; AMM, ammonia; TP, total protein; UN, urea nitrogen. Con group: sow and offspring pigs fed with a basal diet; S-OA group: sow and offspring pigs fed with antibiotics; S-OP group: sow and offspring pigs fed with probiotics; S-OS group: sow and offspring pigs fed with synbiotics. The replicates of four groups at 65 d old were 8. The replicates of the Con, S-OA, S-OP, and S-OS groups at 95 d old were 8, 8, 8, and 7, respectively. The replicates of the Con, S-OA, S-OP, and S-OS groups at 125 d old were 8, 5, 6, and 6, respectively.

## Data Availability

The data presented in the study are included in the article. Further. Inquiries can be directed to the corresponding author.

## Supplementary Materials

The following supporting information can be downloaded at: https://www.mdpi.com/article/10.3390/ijms24087668/s1.
