# Supplementary material for: Sow-Offspring Diets Supplemented with Probiotics and Synbiotics Are Associated with Offspring’s Growth Performance and Meat Quality
Source: Int J Mol Sci. 2023 Apr 21;24(8):7668. doi: 10.3390/ijms24087668 (PMC10144797; doi:10.3390/ijms24087668)
Supplement: Supplementary file 1 [file ijms-24-07668-s001.zip › ijms-2309352-supplementary.pdf]

Table S1. Composition and nutrient levels of basal diets for sows (air-dry basis; %)

Items	Pregnant diet	Lactating diet
Ingredients		
Corn	37.50	66.00
Soybean meal	9.50	25.00
Wheat bran	14.00	5.00
Barley	25.00	
Soybean hull	10.00	
Pregnant premix[1)]	4.00	
Lactating premix[2)]		4.00
Total	100.00	100.00
Nutrient levels[3)]		
Digestible energy (MJ/Kg)	12.55	13.87
Crude protein	12.82	16.30
Crude fiber	4.56	2.87
SID[4)] Lys	0.48	0.75
SID Met+ Cys	0.43	0.51
SID Thr	0.37	0.53
SID Trp	0.13	0.17
Calcium	0.62	0.65
Phosphorus	0.47	0.50

[1)] Pregnant premix provided the following per kg of diet: $CaHPO_4{\cdot}2H_2O$ 10 g, NaCl 4 g, $CuSO_4{\cdot}5H_2O$ 80 mg, $FeSO_4{\cdot}H_2O$ 360 mg, $ZnSO_4{\cdot}H_2O$ 240 mg, $MnSO_4{\cdot}H_2O$ 100 mg, $MgSO_4{\cdot}7H_2O$ 1 g, 1% ICl 50 mg, 1% Na_2SeO_3 36 mg, 1% $CoCl_2$ 16 mg, $NaHCO_3$ 1.4 g, VA 10 000 IU, VD_3 1 800 IU, VE 20 mg, VK_3 2.4 mg, VB_1 1.6 mg, VB_2 6 mg, VB_6 1.6 mg, VB_{12} 0.024 mg, folic acid 1.2 mg, nicotinamide 20 mg, pantothenic acid 12 mg, biotin 0.12 mg, ferrous glycinate 100 mg, choline chloride 1g, phytase 200 mg, fruity 80 mg, and limestone 12 g.

[2)] Lactating premix provided the following per kg of the diet: $CaHPO_4{\cdot}2H_2O$ 10 g, NaCl 4 g, $CuSO_4{\cdot}5H_2O$ 80 mg, $FeSO_4{\cdot}H_2O$ 360 mg, $ZnSO_4{\cdot}H_2O$ 240 mg, $MnSO_4{\cdot}H_2O$ 100 mg, 1% ICl 50 mg, 1% Na_2SeO_3 36 mg, 1% $CoCl_2$ 16 mg, $NaHCO_3$ 1.4 g, VA 10 000 IU, VD_3 1 800 IU, VE 20 mg, VK_3 2.4 mg, VB_1 1.6 mg, VB_2 6 mg, VB_6 1.6 mg, VB_{12} 0.024 mg, folic acid 1.2 mg, nicotinamide 20 mg, pantothenic acid 12 mg, biotin 0.12 mg, Lysine 1.5 g, ferrous glycinate 100 mg, choline chloride 1g, phytase 200 mg, fruity 80 mg, limestone 12 g.

[3)] Nutrient levels were calculated values.

[4)] SID: standard ileum digestible.

Table S2. Composition and nutrient levels of basal diets for weaned Bama mini-pigs (air-dry basis; %)

Items	Prophase diet (35–95 d-old)	Anaphase diet (96–125 d-old)
Ingredients		
Corn	54.92	58.00
Soybean meal	22.00	18.35
Wheat bran	10.13	11.35
Rice bran	8.95	8.30
Premix[1)]	4.00	4.00
Total	100.00	100.00
Nutrient levels[2)]		
Digestible energy (MJ/kg)	13.50	13.42
Crude protein	16.13	14.90
Calcium	0.45	0.44
Total Phosphorus	0.49	0.49
Lys	1.40	1.30
Met + Cys	0.69	0.66
Thr	0.78	0.74

[1)] Premix provided the following per kilogram of diets: enzyme preparation (including phytase, protease, and lipase) 1.2 g, VA 26 000 IU, VD_3 10 000 IU, VE 70 IU, VK_3 10 mg, VB_1 10 mg, VB_2 25 mg, VB_6 10 mg, VB_{12} 0.075 mg, biotin 0.4 mg, folic acid 5 mg, nicotinamide 100 mg, pantothenic 50 mg, choline 1600 mg, flavoring agent 500 mg, edulcorant 300 mg, acidulating agent 5 g, $CuSO_4 \cdot 5H_2O$ 898 mg, $MnSO_4 \cdot H_2O$ 298 mg, $ZnSO_4 \cdot H_2O$ 600 mg, $FeSO_4 \cdot H_2O$ 501 mg, $Ca(IO_3)_2$ 0.9 mg, as Na_2SeO_3 0.7 mg, $CoSO_4 \cdot H_2O$ 1.2 mg, glucose 2.1 g, antioxidants 0.4 g, anti-mildew agent 1 g, Ca (as $CaHPO_4$ and $CaCO_3$) 3.42 g, and P (as $CaHPO_4$) 1.155 g.

[2)] Nutrient levels were calculated values.

Table S3. Primers sequences used for real time-PCR

Genes	GenBank ID	Sequence (5′-3′)	Size (bp)
β-actin	XM_021086047.1	F: GGCACCACACCTTCTACAACGAG	102
		R: TCATCTTCTCACGGTTGGCTTTGG	
MyHCI	NM_213855.2	F: CTGTCCAAGTTCCGCAAGGT	176
		R: CTTTGTTGCGCCCTCAGGAT	
MyHCIIa	NM_214136.1	F: GGACCCCCTGAATGACACAG	149
		R: CGGTCTGGAAGGAAGAACCC	
MyHCIIx	NM_001104951.2	F: TGAGGAAGACCGCAAGAACA	272
		R: GGTCACTTTTGAGCATTTGGATG	
MyHCIIb	NM_001123141.1	F: AGGAGCATCAGCGCCTAATC	119
		R: TCGGGATAGCTGAGACACCA	
MyoD	NM_001002824.1	F: CTATGATGACCCGTGTTTCG	101
		R: AGTGTTCCTCGGGCTTTAGG	
Myf5	NM_001278775.1	F: GGATCAGCAACTCCGAGCAACC	126
		R: GCACATGGTAGATGAGCCTGGAAC	
Myf6	NM_001244672.1	F: GCTCGTGATGACTGCCAAGGAAG	80
		R: CGATGGAAGAAAGGCACCGAAGG	
MyoG	NM_001012406.1	F: AAACTACCTGCCCGTCCACCTC	112
		R: GGTCCCCAGCCCCTTATCTTCC	
*IGF*1	NM_214256.1	F: GACGCTCTTCAGTTCGTGTG	141
		R: CTCCAGCCTCCTCAGATCAC	
FBOX32	NM_001044588.1	F: AAGGGAACTCCTCCAGACC	104
		R: CCATCCGATACACCCACAT	
MSTN	NM_214435.2	F: GCACCAAGCAAACCCCAGAGG	143
		R: AGCACCCACAGCGATCTACTACC	

Note: *MyHCI*, myosin heavy chain I; *MyHCIIa*, myosin heavy chain IIa; *MyHCIIb*, myosin heavy chain IIb; *MyHCIIx*, myosin heavy chain IIx; *MyoD*, myogenic differentiation factor; *MyoG*, myogenin; *Myf5*, myogenic factor 5; *Myf6*, myogenic factor 6; *IGF-1*, insulin-like growth factor-1; *FBOX32*, muscle atrophy Fbox-1 protein; *MSTN*, myostatin.
